# Major Factors Influencing the Size Distribution Analysis of Cellulose Nanocrystals Imaged in Transmission Electron Microscopy

**DOI:** 10.3390/polym13193318

**Published:** 2021-09-28

**Authors:** Hui Qian

**Affiliations:** Nanotechnology Research Center, National Research Council of Canada, 11421 Saskatchewan Drive, Edmonton, AB T6G 2M9, Canada; Hui.Qian@nrc-cnrc.gc.ca

**Keywords:** nanoparticle size distribution, cellulose nanocrystals, negative staining, cryo-TEM ADF-STEM, electron tomography, coffee-ring effect, fractionation

## Abstract

Size distributions of cellulose nanocrystals (CNCs), extracted from softwood pulp via strong sulfuric acid hydrolysis, exhibit large variability when analyzed from transmission electron microscopy (TEM) images. In this article, the causes of this variability are studied and discussed. In order to obtain results comparable with those reported, a reference material of CNCs (CNCD-1) was used to evaluate size distribution. CNC TEM specimens were prepared as-stained and dried with a rapid-flushing staining method or hydrated and embedded in vitreous ice with the plunge-freezing method. Several sets of bright-field TEM (BF-TEM), annular dark-field scanning TEM (ADF-STEM) and cryogenic-TEM (cryo-TEM) images were acquired for size distribution analysis to study the contributing factors. The rapid-flushing staining method was found to be the most effective for contrast enhancement of CNCs, not only revealing the helical structure of single CNCs but also resolving the laterally jointed CNCs. During TEM specimen preparation, CNCs were fractionated on TEM grids driven by the coffee-ring effect, as observed from contrast variation of CNCs with a stain-depth gradient. From the edge to the center of the TEM grids, the width of CNCs increases, while the aspect ratio (length to width) decreases. This fractionated dispersion of CNCs suggests that images taken near the center of a droplet would give a larger mean width. In addition to particle fractionation driven by the coffee-ring effect, the arrangement and orientation of CNC particles on the substrate significantly affect the size measurement when CNC aggregation cannot be resolved in images. The coexistence of asymmetric cross-section CNC particles introduces a large variation in size measurement, as TEM images of CNCs are mixed projections of the width and height of particles. As a demonstration of how this contributes to inflated size measurement, twisted CNC particles, rectangular cross-section particles and end-to-end jointed CNCs were revealed in reconstructed three-dimensional (3D) micrographs by electron tomography (ET).

## 1. Introduction

Cellulose nanocrystals (CNCs) are crystalline particles derived from naturally abundant plant or animal cellulose sources (wood, cotton, tunicate and bacteria, etc.) via strong acid hydrolysis. Depending on the origin of bulk cellulose and acid hydrolysis conditions, the crystallinity, shape and geometric dimensions of extracted CNCs exhibit great variety. In general, CNCs come in rod, ribbon or whisker-like shapes, with lengths ranging from tens of nanometers to several microns and widths ranging from 3 to 50 nm [1,2,3,4]. Compared with bulk cellulose with greater amorphous fractions, CNCs exhibit a higher aspect ratio (length-to-width) with the reactive surface of hydroxyl side groups, a greater axial elastic modulus and unique liquid crystalline properties. These properties, along with their natural abundance and biocompatibility, make CNCs attractive for many industrial applications, such as sustainable energy and electronics, biomedical engineering, water treatment, etc. [5,6,7,8].

CNCs are reminiscent of crystalline regions within elementary fibrils, and the cross-sections of terminating surfaces are either square, rectangle or hexagon [9]. However, CNCs derived following acid hydrolysis display considerable variability in crystallinity and morphology despite being from the same cellulosic source. The processing conditions during the exaction and post-drying of CNCs may result in the variability of CNC products [3,10]. On the other hand, the characterization of products, as a bridging procedure between production and utilization, may also provide inconsistent results due to varying characterization techniques. This variability makes further functionalization and application of CNCs inconsistent. Therefore, the development of consistent, reliable and accurate measurement protocols is critical to understand the various processes required for optimizing CNC production and utilization.

The structure and size distribution of CNCs, as significant physicochemical properties, have been characterized using light scattering and electron microscopy for several decades. Since the early 1950s, transmission electron microscopy (TEM) has been used to reveal the morphology of CNCs extracted from cotton, ramie and bacterial cellulose [1,11]. Samira et al. performed comprehensive characterization using TEM/cryogenic-TEM, atomic force microscopy (AFM) and small- and wide-angle X-ray scattering (SAXS and WAXS) to determine the shape and size distribution of CNCs from several cellulose sources by [4]. However, challenges in CNC size distribution analysis still remain, and a standard analysis protocol has yet to be established, although numerous efforts have been made in several aspects, including analysis methods and material treatment [12,13,14]. Johnston’s team recently reported interlaboratory comparisons of CNC size distributions measured with both TEM and AFM. The results show great variability between the participating laboratories, and a skew-normal distribution method was proposed to accommodate the variability from different datasets [15,16]. Interestingly, the mean width (7.5 nm) measured in TEM is still approximately twice the mean height (3.4 nm) measured in AFM. This may indicate that not all CNC particles are composed of single crystallites, CNC particles are composed of single crystallites in different sizes or CNCs are laterally jointed bundles. More recently, both the width and height of CNCs were measured using AFM images with an internal calibration standard to evaluate the broadening effect of the AFM tip on width measurement. The results show 28% of CNCs with an approximately symmetric transverse cross-section (square) and the remainder with an asymmetric cross-section [17]. However, a validation procedure is required for width measurement in AFM, which may introduce another source of variability from gold nanoparticles used as the internal calibration standard.

Unlike AFM topography images, TEM images are the projection of three-dimensional (3D) objects along the electron-beam direction on a camera or detector as a two-dimensional (2D) image. Therefore, the arrangement and orientation of CNCs on substrate/TEM grids will affect the projected image, which is used for size measurement. As illustrated in the schematic diagram (Figure S1) in the Supplementary Materials, the width measured from a projected image is equal to the height when a rod-shaped particle has a symmetric cross-section. However, with an asymmetric cross-section, they may be different, depending on which side lies on the substrate. Therefore, the measured width distribution from TEM images is a mix of the width and height of 3D particles. In addition to the orientation on the substrate, CNC particle arrangement may also introduce variation in width and length measurements when individual particles cannot be resolved. Furthermore, sessile droplets on TEM grids may cause the CNC particles to be distributed fractionally during drying, leading to an inaccurate size distribution due to the missing representation of particles from certain regions of the TEM grids. In this article, several image sets with different imaging conditions were processed to evaluate the size distribution of CNCs. The major factors influencing the size distribution of CNCs will be discussed in depth based on observations and analysis.

## 2. Materials and Methods

### 2.1. TEM Specimen Preparation

The CNC material used is a National Research Council Canada-certified reference (NRCC), CNCD-1 [12,15,16,17,18]. The CNCs were extracted with sulfuric acid hydrolysis of softwood pulp, followed by neutralization and sodium exchange, purification and spray drying. The CNC aqueous solution was prepared by dispersing dry CNC powders in deionized water with a concentration of 0.02% (*w*/*v*). Vortex or sonication of the CNCs in aqueous solution was performed before preparing TEM specimens.

Continuous and perforated carbon-film-supported TEM grids were treated with glow discharge in air for 15 s at 15 mA current (PELCO easiGlow™, Ted Pella, Inc., Redding, CA, USA) before applying samples. CNC TEM specimens were prepared under four different conditions as described below. The first specimen (Sp1) was CNCs negatively stained with 2% uranyl acetate (UA) on TEM grids. Briefly, a 3 µL droplet of CNC aqueous solution was deposited on an ultra-thin continuous carbon-film TEM grid. Excess solution was blotted off with filter paper from the edge of the grid or the top of the droplet after 10 s incubation. The TEM grid was then tilted 30 to 45 degrees, rapidly washed with two running UA droplets and placed with a 2 µL droplet. After about 30 s of incubation, UA was blotted away with filter paper from the edge of the grids, and a thin layer of stain was left to air-dry on the TEM grids. The second specimen (Sp2) was pristine CNCs deposited on carbon TEM grids with the same preparation procedure as Sp1, except that the droplet incubation time was 60 s without UA staining. The third specimen (Sp3) was negatively stained CNCs on carbon TEM grids with gold nanoparticles as fiducial markers for electron tomography (ET). Colloid gold nanoparticles with a 5 nm diameter (0.1 mg/mL) were deposited on carbon-coated TEM grids and air-dried prior to CNC deposition. The same procedure for Sp1 was then followed to obtain negatively stained CNCs.

The fourth specimen (Sp4) was CNCs embedded in vitreous ice, and the cryo-TEM specimen was prepared using the plunge-freezing method [19]. Briefly, the plunge freezer Dewar/chamber (EMS-002 Rapid Immersion Freezer, Electron Microscopy Sciences, Hatfield, PA, USA) was precooled with liquid nitrogen for 30 min. Compressed ethane gas was liquefied in a Falcon tube surrounded with liquid nitrogen. The liquid ethane was then poured into a cryogen container immersed in a liquid nitrogen chamber with a temperature of −184 °C. The container was ready for use when the ethane was lightly frozen at the bottom and the walls of the container. One 4 µL droplet of CNC aqueous solution was placed on the perforated carbon side of a TEM grid facing up for 2 min. The tweezer securing the TEM grid was then mounted to the releasing anvil. Excess solution was blotted using filter paper from the back side of the TEM grid for 4 s, and the TEM grid was rapidly plunged into the liquid ethane. The frozen CNCs on the TEM grid were kept in liquid nitrogen and transferred for cryogenic-TEM (cryo-TEM) imaging.

### 2.2. Image Acquisition

All bright-field TEM (BF-TEM) and annular dark-field scanning TEM (ADF-STEM) images of CNCs were obtained using a JEOL 2200FS TEM/STEM at a 200 kV accelerating voltage with an in-column Omega energy filter. BF-TEM images with a pixel size of 2048 × 2048 were acquired on a Gatan slow-scan camera with pixel resolution at selected magnifications calibrated with a standard specimen (MAG*I*CAL, Electron Microscopy Science, USA). For imaging CNCs embedded in vitreous ice, the frozen specimen was transferred to a cryo-TEM holder (Gatan Inc., Pleasanton, CA, USA) while being kept below −170 °C. During TEM imaging, the specimen was kept at −180 °C, and the electron beam was blanked between image acquisitions to minimize beam damage on CNCs. The beam dose for each image was 2 e^−^/A^2^. Ten to fifteen TEM images of CNCs from different regions on the TEM grid were acquired at each magnification.

In STEM mode, ADF-STEM images of CNCs were acquired using a 0.5 nm probe with a pixel dwelling time of 10 µs and an image size of 512 × 512 pixels. The pixel resolution was 0.5 nm, which was the same as the scanning step to avoid oversampling between pixels. Approximately 15 ADF-STEM images of CNCs taken from different areas were used for size measurement.

The TEMography^®^ software package (Frontier Inc., Tokyo, Japan) was used for the acquisition of tilt-serial BF-TEM images, fine alignment of 2D images, reconstruction of 3D data, rendering and visualization of CNCs in 3D. The tilt-serial dataset was collected by tilting the holder along the *x*-axis from −70° to +70° with a 2° increment and a defocus value of 1 µm. Gold nanoparticles were used as fiducial markers and focusing objects during image acquisition and fine alignment.

### 2.3. Size Measurement and Distribution Analysis

**Figure 1:**
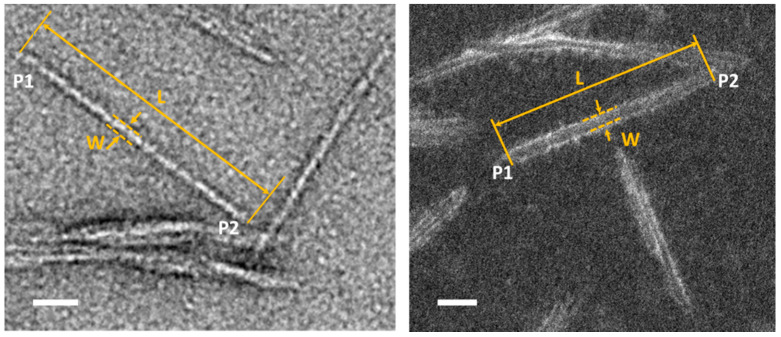
The length (L) is defined as the straight line between the furthest two points (P1 & P2) along the individual CNC particle; the width (W) is defined as the distance between two edges of CNC particle, as indicated in BF-TEM image (**left**) and ADF-STEM image of CNCs (**right**). Scale bars are 20 nm.

ImageJ (Version 1.53c), an open-source software, was used for image processing [20]. Single CNC particles or particles with clear separation outlines were manually selected for size measurement. The size of CNC particles was measured directly from TEM/STEM images. As shown in Figure 1, the length (L) is defined as the distance in a straight line between the two furthest points along a single CNC particle (major axis), and the width (W) (minor axis) is measured at the midpoint of its length unless a particle is clearly asymmetric. An ImageJ macro was used to open sequential images automatically after manual measurement of the length and width of selected CNCs on an active image. Measurement results were exported in a data format suitable for further statistical analysis in OriginPro 2017 or Excel. Approximately 200 particles were measured to give adequate statistical confidence. Histograms of length and width distributions for each dataset were plotted, and Gaussian fitting was applied for descriptive statistics. Box plots labeled with median, mean and one standard deviation (SD) above and below the mean were also used for comparing size distributions in datasets of stained CNCs and CNCs in vitreous ice.

## 3. Results and Discussion

### 3.1. Contrast of CNC Particles in a TEM Image

**Figure 2:**
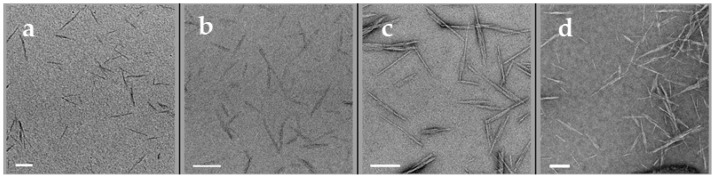
Variation in stain-depth of CNCs shown in BF-TEM images. (**a**) unstained; (**b**) very shallow; (**c**) shallow and (**d**) deep stained CNCs, respectively. Scale bars: 100 nm.

CNCs are low-density (1.6 g/cm^3^), high-aspect-ratio and electron-beam-sensitive biopolymers. The mass contrast of CNC nanoparticles in a normal BF-TEM image is very weak (Figure 2a). To enhance the contrast, conventional negative staining is widely used, and generally, 1–2% aqueous uranyl acetate is used as the staining reagent. However, due to the different staining methods/protocols, inconsistent quality and properties of the supporting films on TEM grids, as well as individual operator skills, staining results vary even for CNCs deposited on the same TEM grids [21]. As shown in Figure S2, a typical region of CNCs stained with 2% uranyl acetate has a gradient stain depth, resulting in CNCs with different contrasts. The very shallow stain cannot cover the overall contour of CNCs, resulting in a weak contrast (Figure 2b). The CNCs in the deep stain appear bright with a fairly uniform dark background (Figure 2d), whereas those in the shallow stain are outlined by the stain (dark outlines), and the helical structure of the CNC particles is revealed clearly (Figure 2c). Despite this stain-depth variation, the rapid-flushing staining method described in the TEM specimen preparation was found to be the most suitable method among all those tested. To ensure that particles selected for image processing and size measurement were as homogenous as possible, regions of CNCs with similar stain depths were chosen as one dataset.

**Figure 3:**
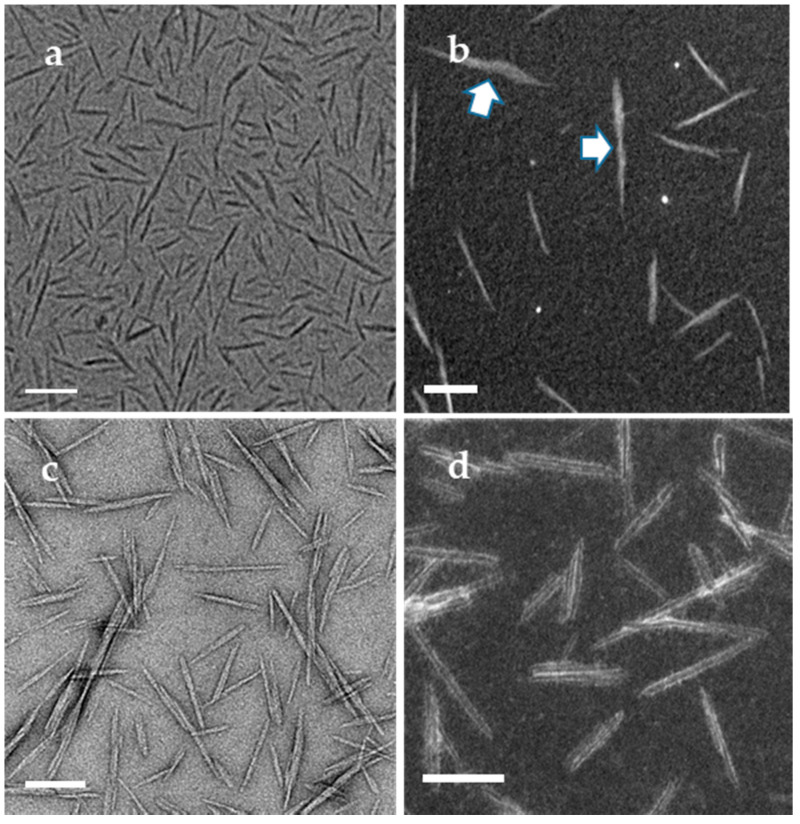
(**a**) under-focused (−3 µm) BF-TEM and (**b**) ADF-STEM images of unstained CNCs; (**c**) BF-TEM and (**d**) ADF-STEM image of stained CNCs. Scale bars: 100 nm.

In addition to heavy-metal staining such as UA, the contrast of CNCs can also be enhanced with other approaches such as electron energy filtering, objective lens defocusing and phase-plate insertion in the BF-TEM mode. As shown in Figure 3a, a zero-loss BF-TEM image of unstained CNCs was taken with a 10 eV energy filter and a defocus of −3 µm, in which the helical-like structure of CNCs was revealed, which indicates that the helical structure in stained CNCs is not an artifact. In the last decade, phase-plate imaging has also been explored in contrast enhancement of soft materials. However, considering the various phase-plate types, phase-shift interpretation and access to instruments with a phase plate, it might be challenging to standardize the size distribution with this approach. In addition to the above approaches of contrast enhancement, STEM imaging is an important method of contrast tuning for polymers and biomolecules [22,23]. The contrast of an ADF-STEM image is related to mass thickness and atomic numbers in the specimen. The pristine CNCs in the ADF-STEM images give high contrast, the individual CNC particles are distinguishable and some CNC particles (pointed by arrows) with nonuniform width along their long axis can be revealed (Figure 3b). On the other hand, electron-beam-induced contaminations can easily build up during scanning and blur the image at high resolution. For CNCs stained with uranyl acetate, edges of CNCs exhibit sharp contrast from the stain with a shallow depth in the ADF-STEM image, as shown in Figure 3d. It is easier to identify the individual CNCs than those in the BF-TEM images (Figure 3c) taken from an adjacent area, even though the helical or twist structure of CNCs is not revealed in the ADF-STEM images, which may not be important for size measurement.

**Figure 4:**
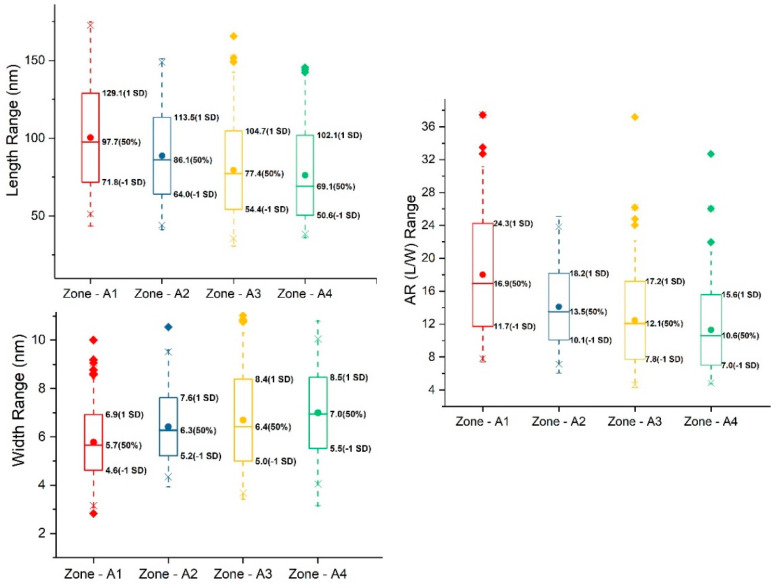
Box plots of length, width and aspect ratio (L/W) distribution of stained.CNCs at zones A1 to A4 with deceasing stain-depth. The line and dot in the box are the median and mean, respectively. The values of the median and one standard deviation (SD) above and below the mean are labelled to the left of boxes.

With the contrast enhancement methods discussed above, sets of BF-TEM and ADF-STEM images of stained and unstained CNCs deposited on carbon TEM grids (Sp1 and Sp2 specimens) were acquired. The length and width of selected CNCs imaged at different conditions were measured, and the descriptive statistics of their distributions are summarized in Table S1. For curiosity, a set of BF-TEM images of CNCs was taken for each of the areas with a gradient stain depth, indicated as Zones A1, A2 and A3 in Figure S2, and the image resolution was 0.41 nm/pixel, 0.41 nm/pixel and 0.29 nm/pixel, respectively. The mean length of CNCs in Zones A1, A2 and A3 is 100.5 nm, 88.8 nm and 79.6 nm, and the mean width is 5.8 nm, 6.4 nm and 6.7 nm, respectively. Moving toward inward areas with a shallow stain (a similar stain depth as in Zone A3), an image set of ADF-STEM for Zone A4 was taken with an image resolution of 0.5 nm/pixel. The mean length and width of CNCs in Zone A4 are 76.3 nm and 7.0 nm, respectively. The histograms of length and width distributions are all included in Figure S3. Box plots of length, width and aspect ratio (length to width) for Zones A1 to A4 are shown in Figure 4. From Zones A1 to A4 with decreasing stain depths, the CNC width increases, while the CNC length and aspect ratio (AR) decrease, which suggests a correlation between size distribution and stain depth or imaging zone. Thus, the question to be answered is: does the stain depth affect the size of CNCs, or are CNC particles distributed differentially or fractionally on the substrate by size during droplet drying?

### 3.2. Dispersion of CNCs Adsorbed on Carbon-Film-Supported TEM Grids

As known, when a sessile droplet containing dispersed solid dries on a solid surface, a characteristic “coffee ring” is formed due to capillary flow toward the droplet edge [24]. In general, during the evaporation of a sessile droplet, the distribution of solids can be affected by capillary flow direction [24], Marangoni flows [25], particle−substrate interactions, particle–particle interaction and particle shape [26]. During TEM specimen preparation, the coffee-ring effect starts once the initial droplet is placed on TEM grids, and the degree of this effect depends on CNC concentration, incubation time, droplet volume and film surface properties. In the initial droplet, rod-shaped particles can be fractionated by particle size along the capillary flow direction when the contact lines of droplets recede on the substrate [27,28]. Particles with a high AR can be orderly arranged near the ring (droplet) edge with their major axis parallel to the contact lines [29]. After a certain incubation time, the initial capillary flow direction is interrupted by shear force from blotting, and the initial droplet is divided into many smaller droplets scattered on the carbon film surface, which might cause redistribution of the CNC particles on the substrate. In subsequent staining, the staining reagent may change the CNC distribution and orientation, but the lateral movement of CNCs should be confined within small areas. After drying, coffee rings might be formed, resulting in gradient thickness of stain. In Specimen Sp1 of stained CNCs, 3 µL of CNC solution and a 10 s incubation time for the initial droplet were used to minimize the coffee-ring effect so that CNCs of all sizes are included for quantitative analysis. However, it is difficult to void the coffee-ring effect completely, as seen in the gradient depth of stain in Figure S2. The results from Zone A1 to Zone A4 with decreasing stain depth, showing that the mean width increases while the mean length and the mean AR decrease, are due to the coffee-ring effect fractionating CNC dispersion on the substrate, instead of different stain thicknesses.

**Figure 5:**
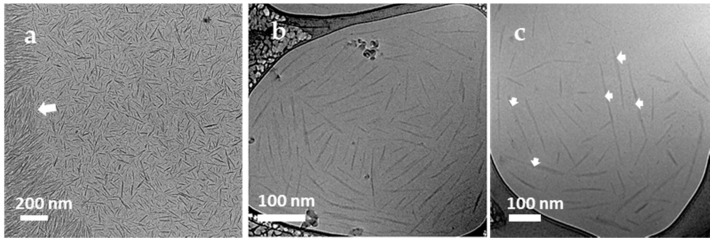
(**a**) coffee-ring formed while unstained CNCs dried on carbon film and more CNCs are concentrated on the edge (white arrow) with well-dispersed CNCs in the center; (**b**) cryo-TEM images of CNCs embedded in vitreous ice taken from the areas near the rim of TEM grids; CNCs lined up parallel to the hole edge and filled across the hole; and (**c**) less ordered CNCs embedded in the ice near the hole edge taken from near the center of TEM grids; possible end-to-end or slightly side-by-side arranged CNCs are pointed by arrows.

To avoid the influence of staining reagent on CNC distribution, unstained CNCs dried on TEM grids (Specimen Sp2) were prepared with the same volume of the initial droplet (3 µL) as Sp1 but with a longer incubation time (60 s). As shown in Figure 5a, well-dispersed CNCs are surrounded by more concentrated CNCs lying along their major axis in a coffee-ring pattern (only part of the ring is shown). The major axis of CNCs was redirected to follow the blotting direction rather than being parallel to the pinned line. The size distribution of CNCs in the center area is shown as Dataset A5 in Table S1. The mean length and width are 85.6 nm and 8.9 nm, respectively. The mean length is comparable to those in Datasets A1 to A4, while the mean width is much larger. Such a large mean width was also reported by Johnston’s team with a consensus distribution of 7.7 ± 2.2 nm [15]. In their case, a 10 µL volume and a 4 min incubation time were used for the initial droplet of CNCs, and an extra washing step with water was added before the subsequent staining of CNCs. The larger droplet volume and longer incubation time made the fractionation and ordered arrangement of CNCs on the substrate driven by the coffee-ring effect more predominant. The washing step may have removed or redistributed some loose CNCs, but the subsequent staining should not affect the dispersion of CNCs already adsorbed on the carbon film surface. Due to the polydispersity of CNCD-1 materials, the fractionated dispersion of CNCs caused by the coffee-ring effect may introduce variability of size distribution when CNCs are imaged and selected from different regions on TEM grids, especially in the radial direction (TEM grid center to edge). The measured mean width of CNCs adsorbed on the center of the TEM grids is larger than that near the edge. Unfortunately, the location of images taken for each laboratory was not specified. In general, areas near the center of the TEM grids would be used for imaging as the default location, likely resulting in biased size distributions.

**Figure 6:**
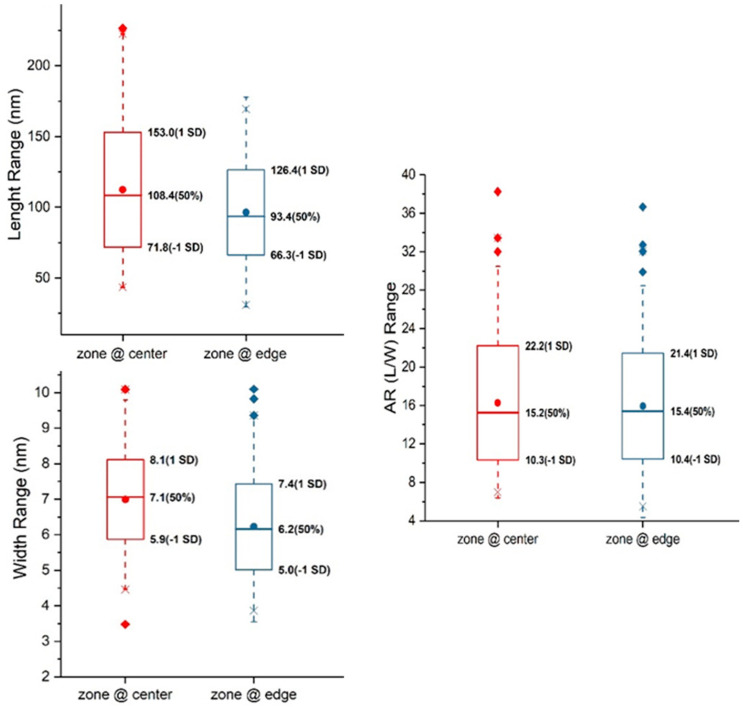
Box plots of length, width and aspect ratio (L/W) distribution of CNCs embedded in vitreous ice near the center and edge of TEM grids. The line and dot in the box is the median and mean, respectively. The values of the median and one standard deviation (SD) above and below the mean are labelled to the left of boxes.

To further prove this coffee-ring effect or the dispersion of CNCs within a droplet, Specimen Sp4 was prepared using the plunge-freezing method to vitrify the CNC aqueous solution without a further drying process. Compared with Specimens Sp1 and Sp2, the incubation time of Sp4 was longer so that most CNCs could settle down within the droplet. Despite blotting from the backside of the droplet, the majority of CNCs near the edge of the droplet were arranged with their major axis parallel to each other (Figure 5b) and dispersed across the entire carbon film hole. CNCs near the center of the TEM grids were less ordered and dispersed than those near the edge of the carbon film hole (Figure 5c). Cryo-TEM images were acquired from regions near the center of the TEM grids as Datasets A6 and near the edge as Dataset A7, respectively. The size distribution in each dataset is summarized in Table S1. The histograms of length and width distributions are shown in Figure S4. The mean width and length with standard deviation for A6 and A7 are 7.0 ± 1.1 nm and 112.4 ± 40.6 nm, 6.2 ± 1.2 nm and 96.4 ± 30.0 nm, respectively. The population of CNCs near the center (A6) has a larger length and width than that near the edge (A7), as shown in the box plots in Figure 6. The ARs in both regions are similar, in contradiction to the dried CNC specimen, Sp1. The blotting direction and TEM grid surface used for the cryo-TEM specimen and the dried CNC specimen were different, which may interfere with the CNC distribution differently during blotting. As illustrated in Figure S5, shear force is induced once the filter paper touches the edge of the continuous carbon-film-coated TEM grid, causing CNCs near the air–water interface to drain along the shear force direction. However, when blotting from the backside of perforated film, the shear force induced flow is through the holes so that CNCs near the air–water interface are drawn within thinned aqueous film rather than being removed [30,31]. CNCs within frozen aqueous film can be dispersed at different heights along the beam direction, which is different from CNCs drying on continuous film after blotting. As shown in Figure 5c, the larger particles marked by arrows might be composed of multiple end-to-end or slightly side-by-side CNC particles or overlapped projections from two particles embedded in different vertical positions, contributing to size inflation for both length and width. 

### 3.3. Orientation of CNCs on Carbon-Film-Supported TEM Grids

**Figure 7:**
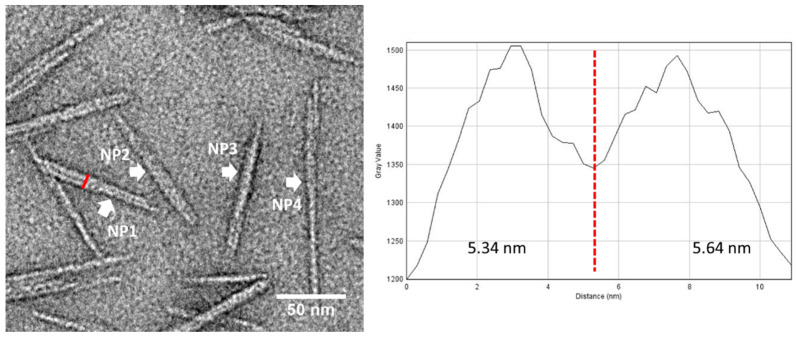
**Right**: Aggregated CNCs with different orientations marked by arrows (NP1, NP2, NP3 & NP4) on carbon film supported TEM grids. **Left**: The line profile of NP1 composed of two laterally jointed CNCs with width of 5.34 nm and 5.64 nm, respectively.

As discussed above, CNCs on TEM grids could be fractionated during TEM specimen preparation, resulting in variability of size distribution when CNCs images are taken from different regions in the radial direction. In addition to the fractionated dispersion, the orientation of CNCs on the substrate also has a significant influence on size measurement from TEM images, as illustrated in Figure S1. In the CNCD-1 materials, about 28% of CNCs are particles with a square or a symmetric cross-section, while the remainder are asymmetric with one axis 2–3 times longer than the other, as analyzed in AFM [17]. When these asymmetric particles adsorb on the substrate surface with random orientations, the width distribution measured from TEM images may have large heterogeneity, as the TEM images are mixed projections of width and height from the minor-axial side of CNCs. Due to the strong hydrogen bond, CNCs tend to be laterally or twistedly jointed, and the widest side is normally the preferred orientation on the substrate. Figure 7 shows a typical example of aggregated CNCs in one region, which are either in a lateral (NP1, NP2), vertical and lateral (NP3) or twisted arrangement (NP4). NP3 and NP4 may be composed of more than two CNCs, and the width of NP4 along the long axis is not uniform. All these arrangements and orientations of CNCs on the substrate cause the variability of size measurement both in TEM and AFM. With proper staining of CNCs, the separation line between laterally jointed CNCs can be resolved in TEM. As shown in the line profile of NP1 in Figure 7, two particles with widths of 5.34 nm and 5.64 nm are well revealed and resolved. However, for unstained CNCs dried or embedded in vitreous ice or overstained CNCs, the separation between two CNC particles may not be revealed, resulting in inflated width measurement in TEM. In AFM, due to its limited resolving power in the lateral direction, it is difficult to resolve or separate laterally jointed CNCs. Therefore, the mean width measured with AFM may be overestimated when the orientation of CNCs is on the wider side. When CNC particles are arranged end-to-end or slightly side-by-side as illustrated in Figure S1, the length measurement will be affected if the separation cannot be resolved in either TEM or AFM.

**Figure 8:**
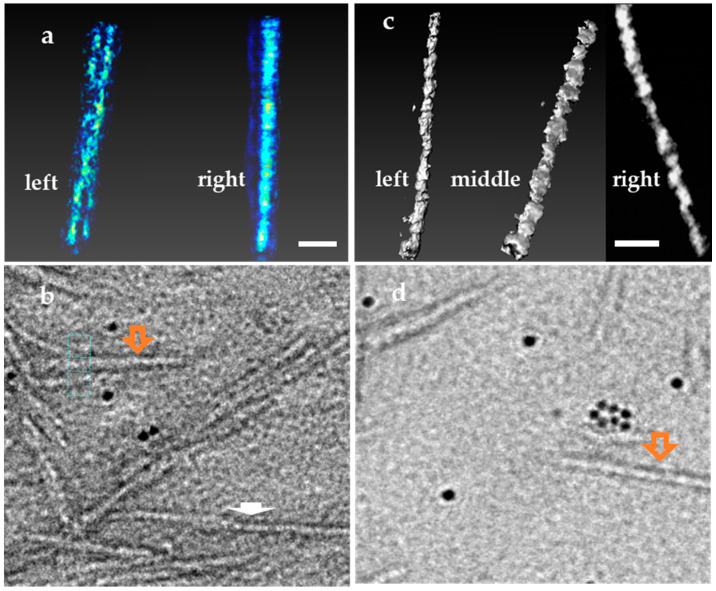
Geometrically twisted CNCs bundles and ribbon-liked CNCs coexists with rod-like CNCs (Sp3 specimen). Scale bars 10 nm. (**a**) Reconstructed 3D volume view of two helical CNCs twisted with an angle (left) and overlapped view (right); (**b**) 2D BF-TEM image of the twisted CNCs (marked as orange arrow) used for 3D reconstruction; (**c**) Reconstructed 3D isosurface of a ribbon-like CNC viewed at an angle with the narrowest width (left), widest width (middle) and the slice view (right); (**d**) BF-TEM image of the CNC used for 3D reconstruction (marked as orange arrow).

**Figure 9:**
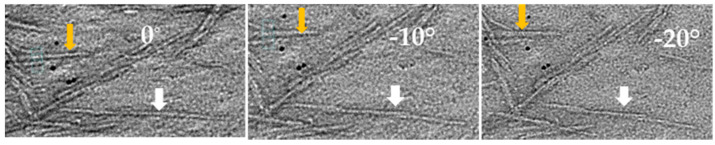
The end-to-end arranged CNC particles is shown as one particle in BF-TEM (**Left**); as two particles (**Middle**) tilted at 10° along long-axis; and as three particles tilted at 20° (**Right**).

To reveal particle orientation and arrangement on the substrate, typical regions of CNC particles on Specimen Sp3 were used for ET. Figure 8a shows a reconstructed 3D micrograph of two geometrically twisted CNCs, which are identified as one individual CNC particle (marked with an orange arrow) in the BF-TEM image (Figure 8b). These two CNCs are screw-like or helical along the long axis, as revealed in the reconstructed volume views. The length and width of these two CNCs measured from the reconstructed 3D data are 57 nm and 3.5 nm and 60 nm and 3.8 nm, respectively. Moreover, in the 2D TEM image (Figure 8b), the width measured for this twisted particle is 5.7 nm, which is used for width distribution analysis. In the same field of view, there is another particle marked with a white arrow. Tilting the particle along the long axis by about 20 degrees showed the particle is actually composed of three end-to-end-connected single CNC particles (Figure 9). Thus, the length measurement for size distribution will give a larger value.

In addition to the particle–particle interactions and arrangement on TEM specimens, the intrinsic shape of CNC particles also contributes to size measurement variability. Ribbon-like CNCs marked with an orange arrow in Figure 8d coexist in the same materials, as shown in Figure 8c of a typical reconstructed 3D isosurface view. The reconstructed 3D data reveal that the CNC particle is helical-like, and its width is not uniform along the long axis. The length, height (thickness) and width of this particle are 60 nm, 3.6 nm (left in Figure 8c) and 5.7 nm (right in Figure 8c), respectively. Thus, this asymmetric or rectangular cross-section suggests that CNC particles with a large width may be composed of more than one single crystallite.

Therefore, the shape, geometrical variation and orientation of CNCs on the substrate all contribute to the size measurement. The size measured from 2D images may not be the true parameter of CNC particles; rather, it is the average size of the projected images of all possible shapes and geometrical arrangements of CNCs deposited on the substrate, resulting in large variability of size distribution.

## 4. Conclusions

High-quality CNC images with good contrast and resolution can mitigate human bias in particle selection and size measurement. The traditional method of staining with heavy metal to enhance contrast for soft materials or biopolymers is still a very effective approach for CNCs. The rapid-flushing staining method has demonstrated that it can reveal the helical structure of single CNCs and separate laterally jointed CNC particles (Figure 2c,d, Figure 3c and Figure 7). It can also reduce the preferential orientation of CNCs on the substrate by running droplets. AFD-STEM imaging is also a good approach for contrast enhancement, as shown in Figure 3, revealing the pristine structure of CNCs. To overcome beam-induced contamination and damage in future work, cooling the TEM holder at liquid nitrogen temperature can be performed when imaging unstained CNCs in ADF-STEM. ADF-STEM images of CNCs with a shallow stain depth give a sharp contrast of CNC particle edges, which may be helpful in particle identification and measurement.

Ideal statistical analysis of size distribution should include CNC particles of all sizes with equal representation. Due to the polydispersity of CNCs and their propensity to agglomerate, it is very challenging to obtain well-dispersed single CNC particles across TEM grids. During TEM specimen preparation, CNC particles could be fractionated across the substrate due to the coffee-ring effect, as seen in dried and hydrated cryo-TEM specimens. Droplet volume, drop-casting incubation time and substrate surface properties will affect the degree of fractionation. A smaller droplet volume and a shorter incubation time are recommended to minimize the coffee-ring effect while preparing CNCs dried on TEM grids. CNCs from different regions of TEM grids, especially in the radial direction, are recommended to be imaged and analyzed for size distribution. Specimen Sp1 is a typical example of fractionated CNCs on a substrate. The size distribution was obtained using the combined datasets from Zones A1 to A3, giving a mean width and length of 6.3 ± 1.3 nm and 89.9 ± 26.2 nm, respectively. Histograms of length and width and descriptive statistics are shown in Figure S6. Compared with the reference values in the certificate (mean width of 7.3 ± 1.8 nm and length of 87.0 ± 35 nm), the length distribution agrees well, but not for the width distribution. The higher mean width of the reference is likely due to lower CNC representation from the edge of droplets with a lower mean width in the measurement sampling, as the center of the droplet is typically the default starting measurement location when TEM grids are loaded inside the instrument. In addition to fractionation, the arrangement and orientation of CNC particles on the substrate significantly affect the size measurement when CNC aggregates cannot be resolved in images. The large mean width in Dataset A5 of unstained CNCs indicates that a large population of laterally jointed or twisted CNCs lying with the widest side on the substrate was included in the analysis. Furthermore, when a large CNC population with an asymmetric cross-section coexists with a symmetric population, the random CNC orientations on the substrate contribute to the variability of the width distribution because the measured width in the TEM images contains the width and height of 3D CNC particles.

In short, to obtain an accurate size measurement of CNCs from TEM images, high-quality CNC TEM specimens and images are essential. To avoid unintended bias of size distribution, imaging and analyzing CNCs from all regions across the TEM grids is recommended. For future work, correlated AFM and TEM imaging can be explored for size distribution analysis of CNCs deposited on the same substrate, which may provide more insight into cross-section shape distribution.

## Data Availability

The data presented in this study are available on request from the corresponding author.

## Supplementary Materials

The following are available online at https://www.mdpi.com/article/10.3390/polym13193318/s1, Figure S1: Schematics of the transverse cross-section, orientation on substrate and projected image on camera of rod-shaped nanoparticles and the possible particle–particle arrangements, Figure S2: Stain-depth variation of CNCs deposited on TEM grid, Table S1: Length and width distributions measured from BF-TEM, ADF-STEM and cryo-TEM images of CNCs, Figure S3: Histograms of length, width and aspect ratio distributions of CNCs measured from images taken in Zones A1, A2 and A3 of stained CNCs on TEM grids (Specimen Sp1), Figure S4: Histograms of length and width distributions for unstained CNCs from TEM specimen Sp2 and cryo-TEM specimen Sp4, Figure S5: Initial droplet of CNC aqueous solution on continuous carbon-film-coated TEM grids and perforated carbon-film-coated TEM grids, Figure S6: Size distribution of CNCs by combining Datasets A1, A2 and A3 measured from Specimen Sp1.
