# Supplementary material for: Major Factors Influencing the Size Distribution Analysis of Cellulose Nanocrystals Imaged in Transmission Electron Microscopy
Source: Polymers (Basel). 2021 Sep 28;13(19):3318. doi: 10.3390/polym13193318 (PMC8513063; doi:10.3390/polym13193318)
Supplement: Supplementary file 1 [file polymers-13-03318-s001.zip › polymers-1387119-supplementary.pdf]

polymers

MDPI

Supplementary Materials

Major Factors Influencing the Size Distribution Analysis of Cellulose Nanocrystals Imaged in Transmission Electron Microscopy

Hui Qian [1,*]

[1] Nanotechnology Research Center, National Research Council of Canada, 11421 Saskatchewan Drive, Edmonton, AB, Canada T6G 2M9
* Correspondence: Hui.Qian@nrc-cnrc.gc.ca

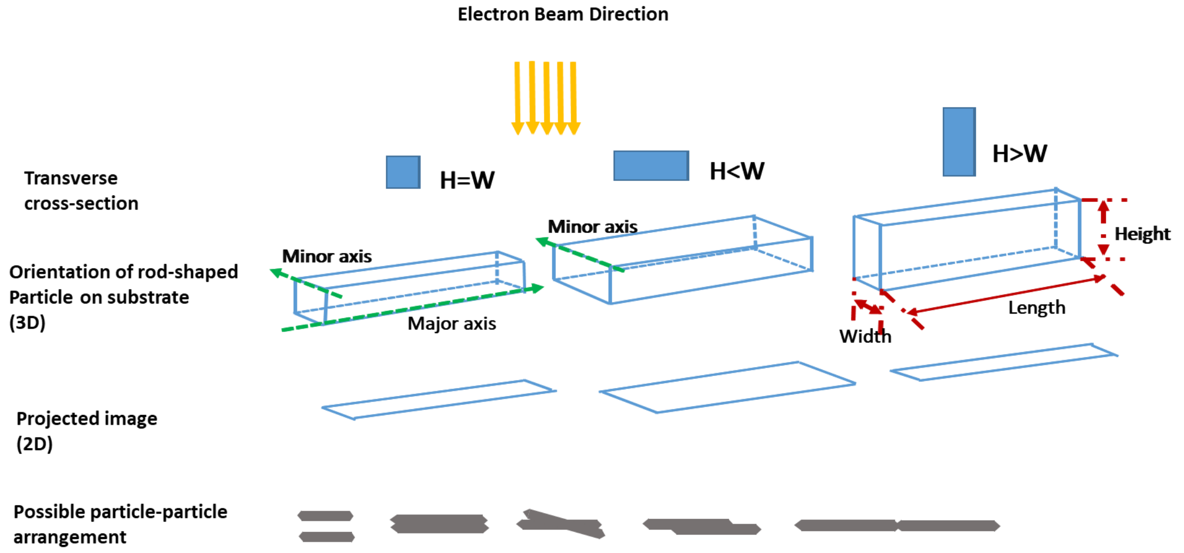

Figure S1. Schematics of the transverse cross-section, orientation on substrate and projected image of rod-shaped nanoparticles and the possible particle-particle arrangements.

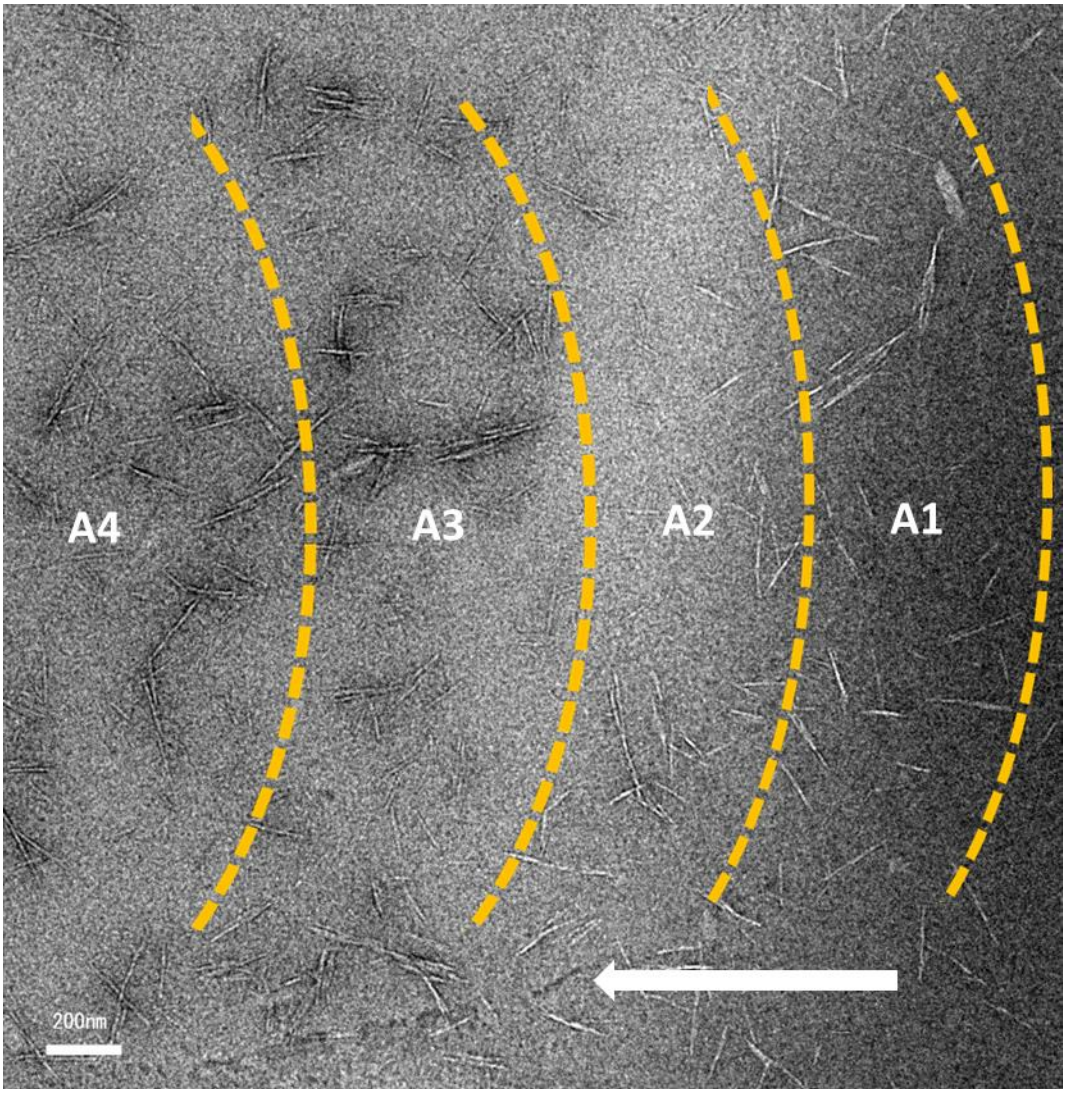

Figure S2. Variation of stain-depth of CNCs deposited on TEM grid. The arrow direction shows the stain depth from deep to shallow, marked as zone A1 to A4.

Table S1. Length and width distributions measured from BF-TEM, ADF-STEM and Cryo-TEM images of CNCs. SD: standard deviation.

Dataset	Number of CNC particles	Imaging Mode	CNCs TEM specimen	Length (L) (nm)		Width (W) (nm)		Aspect ratio (L/W)	Image resolution (nm/pixel)
				mean	SD	mean	SD		
A1	217	BF-TEM	Sp1: Negatively stained –deep	100.5	28.6	5.8	1.2	17.3	0.41
A2	217	BF-TEM	Sp1: negatively stained-shallow	88.8	24.7	6.4	1.2	13.9	0.41
A3	166	BF-TEM	Sp1:Negatively stained-shallow	79.6	25.2	6.7	1.7	11.9	0.29
A4	149	ADF-STEM	Sp1:Negatively stained - shallow	76.3	25.7	7.0	1.5	10.9	0.50
A5	144	BF-TEM (defocus)	Sp2:Unstained air dried	85.6	21.0	8.9	1.6	9.7	1.00
A6	181	Cryo-TEM (BF-TEM)	Sp4:Embedded in vitreous ice	112.4	40.6	7.0	1.1	16.1	0.39
A7	217	Cryo-TEM (BF-TEM)	Sp4:Embedded in vitreous ice	96.4	30.0	6.2	1.2	15.5	0.39

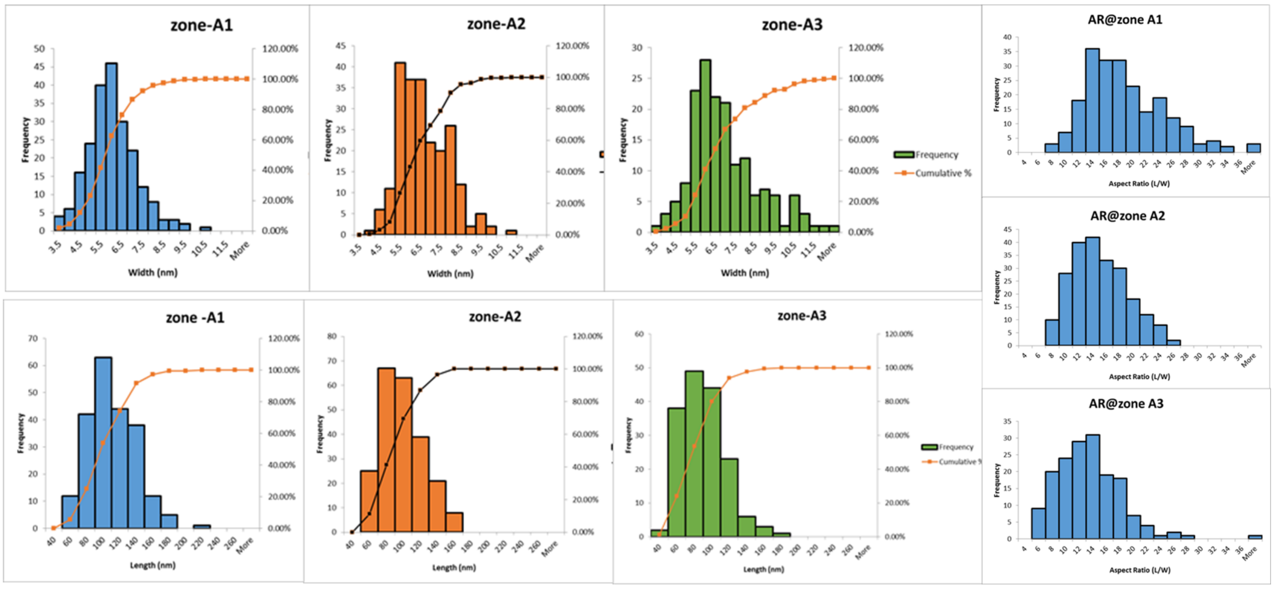

Figure S3. Histograms of length, width and aspect ratio distributions of CNCs measured from images taken in zone A1, A2 and A3 of stained CNCs on TEM grids (specimen Sp1).

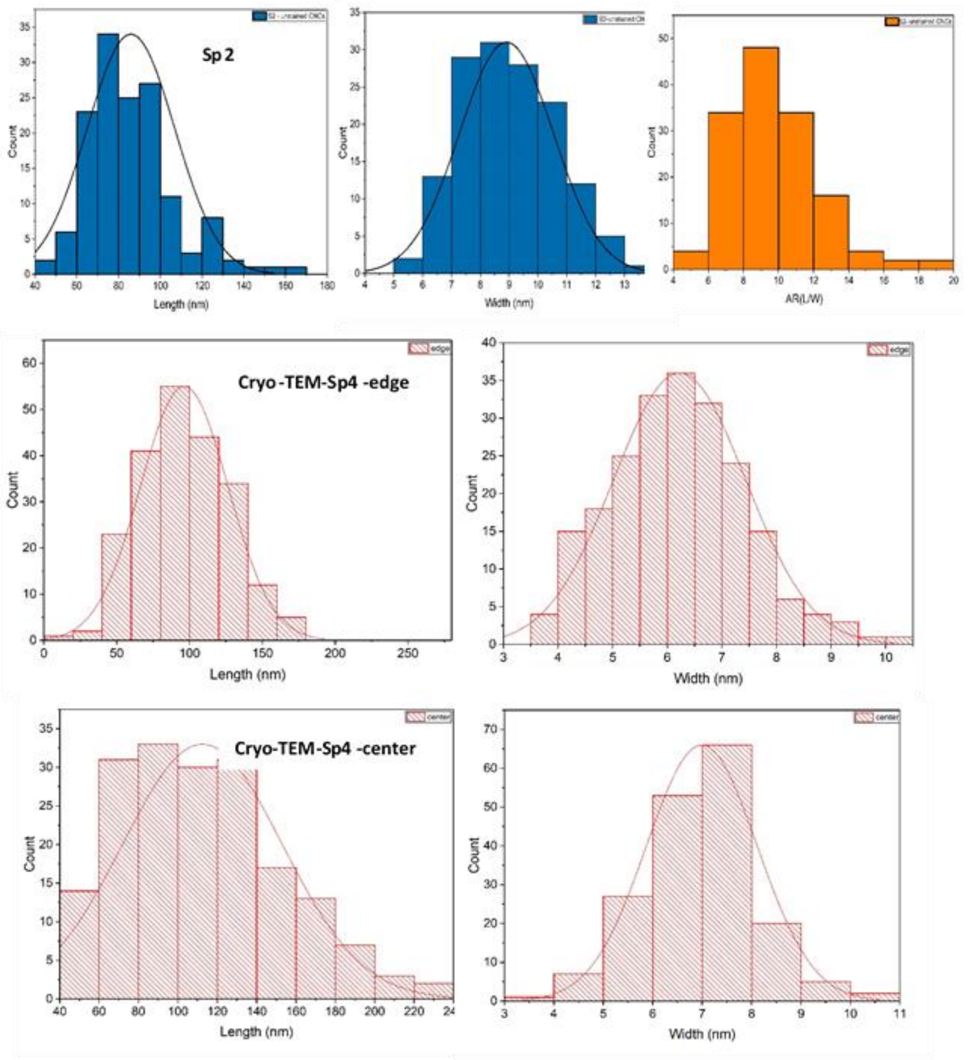

Figure S4 Histograms of length and width distribution for unstained CNCs from TEM specimen Sp2 and cryo-TEM specimen Sp4.

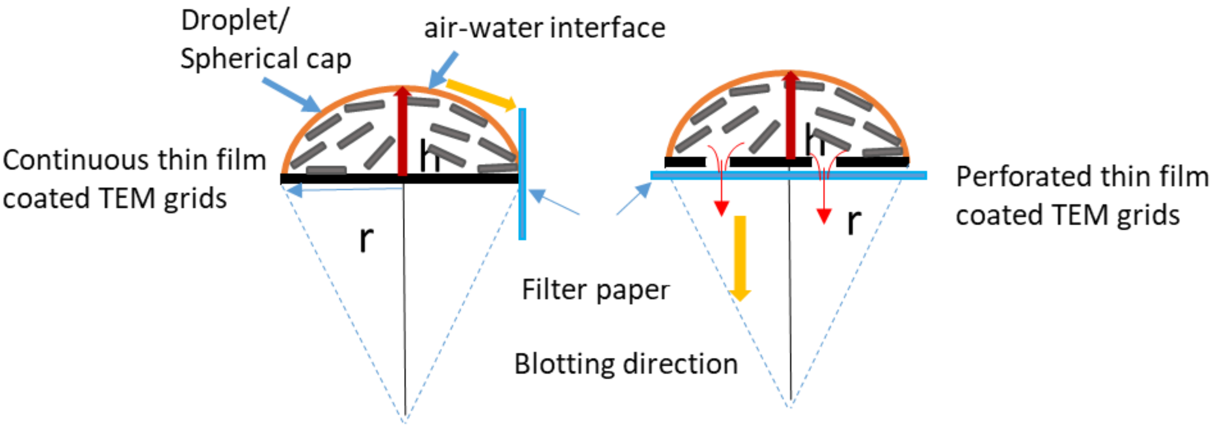

Figure S5. Initial droplet of CNCs aqueous solution on continuous carbon film coated TEM grids (Left) and perforated carbon film coated TEM grids (Right). Blotting directions are indicated by yellow arrows. Radius of spherical cap of droplet is the same and fixed at 1.5 mm. Height (h) is depending on the volume of droplet used.

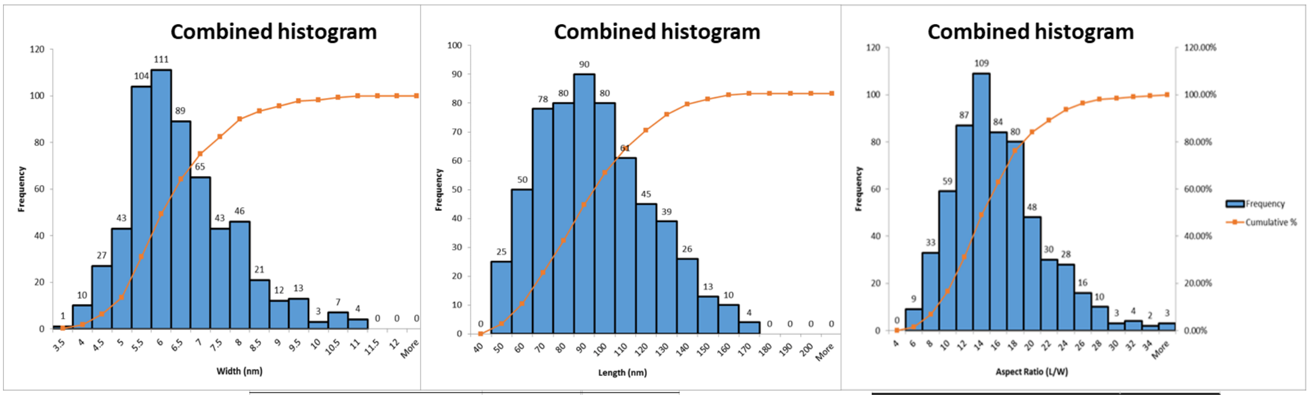

descriptive statistics	*Width*	*Length*
Mean	6.26	89.88
Standard Error	0.05	1.07
Median	6.03	87.37
Mode	5.90	87.74
Standard Deviation	1.32	26.23
Minimum	3.42	40.28
Maximum	10.85	161.95
Count	599.00	599.00
Confidence Level(95.0%)	0.11	2.10

AR(L/W)	
Mean	14.78
Standard Error	0.20
Median	14.05
Mode	14.00
Standard Deviation	5.00
Minimum	4.28
Maximum	31.05
Count	599.00
Confidence Level(95.0%)	0.40

Figure S6. Size distribution of CNCs by combining the datasets of A1, A2 and A3 measured from specimen Sp1. Several data points with value < 3nm and > 11nm in width and large value >180 nm in length were removed from the combined set.
